# Our London Letter

**Published:** 1893-01

**Authors:** Henry Cove


### Correspondence.

### LONDON LETTER.

The weather is an important factor for good or evil in this country, so fickle is the climate, but fortunately it brings good to some, and consequently physicians have no need to worry at the increase of business at this time. In many instances practitioners have found it necessary to employ assistance, so that fewer unemployed qualified men are to be met with at the present time than is usual, but it cannot be denied that the prospects of the coming generation of doctors are not particularly bright. It is a mistake to put a young man to a profession for which he has no liking, simply because his father follows the occupation, but unfortunately this is too frequent an occurrence in this country. The opportunities for a doctor to distinguish himself are much better now then ever they were, and, no doubt, many young and promising physicians have set their heart on doing so, but nevertheless, we seem to be as far as ever from the solution of certain phases of different forms of disease. The recent cholera epidemic afforded ample scope for scientific research, the benefits to the entire human race to be derived from a preventative and cure, would have alone been a handsome reward; but as yet nothing reliable has come to light, although many experiments, still progressing, may lead to some satisfactory result.

Edinburgh University has been the alma mater of many distinguished men, and although the principles of the college are rigidly conservative, the authorities have been obliged to bow (gracefully?) to the exigencies of the time. The lady medical student is now a common sight at the Royal Infirmary of that City, and the number shows signs of increasing. A special ward, medical and surgical, is set apart for their use exclusively, and they are allowed to attend some of the cliniques on special subjects with the men. Some little awkwardness exists, as it will do whenever anything new, especially of this nature, is introduced into an old institution, and our professor insists on the female portion of his hearers occupying the very back bench in the room. Most of the men have sufficient good manners to prevent their turning round.

The present is esentially an age of athletics, and the epidemic of foot-ball often leads to the employment of a doctor, who is usually rewarded with a handsome fee, most of the clubs not being pinched by a lack of funds. However, there should be moderation in all things, as the other day a man exerted himself until his heart failed and he died from syncope, while in another match on the same day, a youth was so violently handled that he died from his injuries. On both occasions was a physician present, but the sufferers were beyond the usual methods of treatment.

The system of practitioners supplying their own medicines has long been popular in the Lancashire districts; and in all rural communities this now appears to be spreading. Some doctors have stated that they object strongly to the idea of depriving any houses of their means of livelihood, as the druggists must of course suffer, but they are bound to do so to support themselves, and their patients seem to like it better. In some districts it is most difficult to get in the customary fees, but several doctors who supply their physic say that these are much more easily collected when medicine figures in bill, especially with the illiterate classes, as these latter never can see that they have received value for their money when only medical advice is given. All businesses appear to be in a transitive stage and the medical profession is not an exception.

HENRY COVE.

